# Two- and Three-Dimensional Modeling and Simulations of Grain Growth Behavior in Dual-Phase Steel Using Monte Carlo and Machine Learning

**DOI:** 10.3390/ma16247536

**Published:** 2023-12-06

**Authors:** Fei Sun, Ayano Kita, Toshio Ogawa, Ta-Te Chen, Yoshitaka Adachi

**Affiliations:** 1 Department of Material Design Innovation Engineering, Nagoya University, Furo-cho, Chikusa-ku, Nagoya 464-8603, Japan; kita.ayano.n9@s.mail.nagoya-u.ac.jp (A.K.); chen.ta.te.w8@f.mail.nagoya-u.ac.jp (T.-T.C.); 2 Department of Mechanical Engineering, Aichi Institute of Technology, 1247 Yachigusa, Yakusa-cho, Toyota 470-0392, Japan; ogawa.toshio@aitech.ac.jp

**Keywords:** grain growth, dual-phase steel, Monte Carlo, machine learning

## Abstract

Dual-phase (DP) steel has been widely used in automotive steel plates with a balance of excellent strength and ductility. Grain refinement in DP steel is important to improve the properties further; however, the factors affecting grain growth need to be well understood. The remaining problem is that acquiring data through experiments is still time-consuming and difficult to evaluate quantitatively. With the development of materials informatics in recent years, material development time and costs are expected to be significantly reduced through experimentation, simulation, and machine learning. In this study, grain growth behavior in DP steel was studied using two-dimensional (2D) and three-dimensional (3D) Monte Carlo modeling and simulation to estimate the effect of some key parameters. Grain growth can be suppressed when the grain boundary energy is greater than the phase boundary energy. When the volume fractions of the matrix and the second phase were equal, the suppression of grain growth became obvious. The long-distance diffuse frequency can promote grain growth significantly. The simulation results allow us to better understand the factors affecting grain growth behavior in DP steel. Machine learning was performed to conduct a sensitivity analysis of the affecting parameters and estimate the magnitude of each parameter’s effects on grain growth in the model. Combining MC simulation and machine learning will provide one promising research strategy to gain deeper insights into grain growth behaviors in metallic materials and accelerate the research process.

## 1. Introduction

Metallic structure factors that control the mechanical properties of dual-phase (DP) steel include the volume fraction and distribution behavior of each phase, grain morphology and size, etc. Grain size control is an important research topic in materials science and engineering since grain size distribution strongly affects the mechanical properties of many alloys. Regarding grain size, the finer the grain size, the better the mechanical properties of the material, according to the Hall–Petch relationship [1,2]. Great efforts have been made to form a fine grain structure and suppress grain growth [3,4,5,6]. Grain growth is a process through which the average grain or crystal size in a fully crystalline material increases with time. This grain size increase occurs through competition in which some grains shrink and disappear while other grains grow to fill the volume formerly occupied by the shrinking grains [7]. Experimental approaches to suppress grain growth in DP steels have been carried out for a long time to improve the properties of the material itself [8]. Experimental research on grain refinement in DP steel has focused on elucidating the mechanism of grain growth [9]. However, the affecting factor determining grain growth cannot be identified through experiments.

Computer simulations have been used to conduct detailed analyses that consider various histological factors included in polycrystals [10]. Experiments, computer simulations, and machine learning have become a trinity in recent years. Integrated materials research is being actively conducted, and materials informatics, which combines materials science and data science, is attracting attention. Computer simulation of grain growth behavior has been studied in the early years [11,12,13,14,15,16,17,18]. The Monte Carlo (MC) computer simulation technique is one of the most promising methods for detailed information on the topology and kinetics of microstructure evolution and retains a number of features that derive from the closely related Ising and Potts models [14,15]. The potential for nonuniform grain boundary mobility to act as a persistence mechanism for abnormal grain growth was investigated using MC Potts model simulations [16]. In addition, grain growth in the weld heat-affected zone of multipass welds in 314 stainless steel was successfully studied using the MC model [17]. A three-dimensional (3D) MC model with nucleation in each MC step was applied to simulate the grain growth during the friction stir welding process [18]. In these studies, MC simulation results and experimental data on the radii of austenite and ferrite grains in dual-phase low-alloy steel and duplex stainless steel agree well with the proposed relation using two-dimensional (2D) simulation [19]. The grain growth process resulting from local grain boundary movement is modeled using MC simulations that can reproduce the actual structure well. It has become possible to reproduce the temporal changes and morphology of grain growth using 3D simulation [20]. Grain growth is driven by the decrease in interfacial energy.

Due to the aforementioned trends in the development of MI, there is a need for efficient data acquisition of material structures, and these conventional studies are again attracting attention. Therefore, this study aims to use the MC method to construct a grain growth behavior model of DP steel and propose a research strategy to speed up the material structure analysis of steel materials combined with machine learning. Two-dimensional and 3D MC models will be developed, and the corresponding simulations will be performed to compare the results and differences for quantitative clarification. Furthermore, based on the simulation results, performing machine learning will clarify the factors governing grain growth. Machine learning could be performed to conduct a sensitivity analysis of the affecting parameters and estimate the magnitude of each parameter’s effects on grain growth in the model. Combining MC simulation and machine learning will provide one promising research strategy to gain deeper insights into grain growth behaviors in metallic materials and accelerate the research process.

## 2. Monte Carlo Simulation and Machine Learning

**Figure 1:**
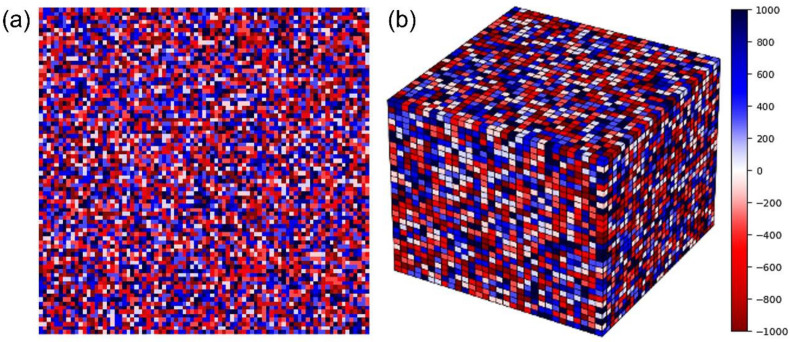
(**a**) Two-dimensional and (**b**) 3D models representing the initial microstructure in the Monte Carlo simulation. The integers in the color scale indicate the crystal orientation of the cells. The positive and negative integers represent the matrix and the second phase, respectively.

Figure 1a,b shows the developed models, indicating the initial microstructures for two-dimensional (2D) and three-dimensional (3D) simulations, respectively. In Figure 1a, the microstructure is generated based on a 2D 100 × 100 array of square cells for the 2D simulation. In Figure 1b, the microstructure is generated based on a 3D 30 × 30 × 30 array of square cells for the 3D simulation. Positive integers from 1 to 1000 and negative integers from −1 to −1000 were randomly assigned to the matrix and the second-phase cells, respectively. These integers correspond to crystal orientations; a collection of unit cells with the same integer represents a grain. The proportion of negative numbers was determined according to the volume fraction of the second phase.

Grain growth behavior simulations were performed by considering two elementary processes: grain/phase boundary movement and Ostwald growth accompanied by long-distance diffusion [21]. The boundary energy of a random cell is expressed as follows.

(1) $$E_{A}=\sum_{A\neq B}^{k}\left\{E_{gb1}\left(1-\delta_{S_{A}S_{B}}\right)\varepsilon_{AB}+E_{pb}\left(1-\varepsilon_{AB}\right)\right\}\;\left(S_{A}>0\right)$$

(2) $$E_{A}=\sum_{A\neq B}^{k}\left\{E_{gb2}\left(1-\delta_{S_{A}S_{B}}\right)\varepsilon_{AB}+E_{pb}\left(1-\varepsilon_{AB}\right)\right\}\;\left(S_{A}<0\right)$$

Here, $E_{gb1}$, $E_{gb2}$, $E_{pb}$ are the matrix grain boundary energy, the second-phase grain boundary energy, and the matrix/the second phase boundary energy, respectively. $S_{A}$ and $S_{B}$ are the crystal orientations of cell A and the *k*th neighboring cell B, respectively. $\delta_{S_{A}S_{B}}$ is set to 1 when $S_{A}=S_{B}$ and 0 when $S_{A}\neq S_{B}$. $\varepsilon_{AB}$ is set to 1 when cell A and B belong to the same phase and 0 when they belong to different phases.

Growth through grain/phase boundary migration proceeds by changing the orientation of a randomly selected cell or by exchanging the orientation of a selected cell with its neighboring cell, so the boundary energy calculated by Equations (1) and (2) is minimized. If the energy of the one cell of interest is lower or the same after the orientation change or swap, then the swap operation is valid. In other cases, the orientation of the target cell is restored to its initial orientation. The difference between growth due to grain/phase boundary movement and long-distance diffusion is that in the former, the focused cell attempts to change and exchange orientation with adjacent cells, whereas in the latter, the focused cell attempts to change and exchange orientation with distant cells. Table 1 lists some parameters for the 2D and 3D simulations. The boundary energy ratio of $E_{gb1}:\;E_{gb2}:E_{pb}=1:1:3$ indicates the grain boundary energy in the matrix and the second phase is the same, but smaller than the matrix/the second phase boundary energy. The ratio of $E_{gb1}:\;E_{gb2}:E_{pb}=1:1:1$ indicates grain boundary energy in the matrix and the second phase is the same as the matrix/the second phase boundary energy. The ratio of $E_{gb1}:\;E_{gb2}:E_{pb}=3:3:1$ indicates the grain boundary energy in the matrix and the second phase is the same, but greater than the matrix/the second phase boundary energy. The long-distance diffusion frequency (LDDF) is 0, 0.5, and 1. LDDF of 1 indicates the growth by long-range diffusion was attempted in all loops. When all sites are updated, that is, several trials of the model size, the unit time is 1 MC, which is taken as the time parameter.

Machine learning was performed using the simulation results to conduct forward and sensitivity analysis based on an Artificial Neural Networks (ANN)-created model. Bayesian optimization was used to optimize the number of nodes and hyperparameters of the middle layer in the neural network. The input was the six variations used for simulation, as shown in Table 1, and the output was the average grain size. Based on the simulation results, 90% of the data was used as training data and 10% as test data to construct the model. Shiny MIPHA was used for model construction and analysis [22]. Sensitivity analysis quantifies the influence of input variables on output. In this study, the connecting weight algorithm [23] was used for the sensitivity analysis. The influence of hidden neuron *h* on an input neuron *i* is expressed by the following equation:

(3) $$S_{ih}=\frac{\left|w_{ih}\right|}{\sum_{r=1}^{n}\left|w_{ih}\right|}\;h=1,\;2,\ldots ,h$$

where *w* is the weight coefficient. The influence of input neuron *i* on output neuron *o* is expressed by:

(4) $$t_{\left(i\right)o}=\sum_{h=1}^{h}\left[S_{ih}\times \left|w_{ho}\right|\right]$$

The value $t_{\left(i\right)o}$ represents the magnitude of each parameter’s effects. The larger the value, the more influence the parameter has on the model.

## 3. Results and Discussion

### 3.1. Monte Carlo Simulation and Affecting Factors of Grain Growth

**Figure 2:**
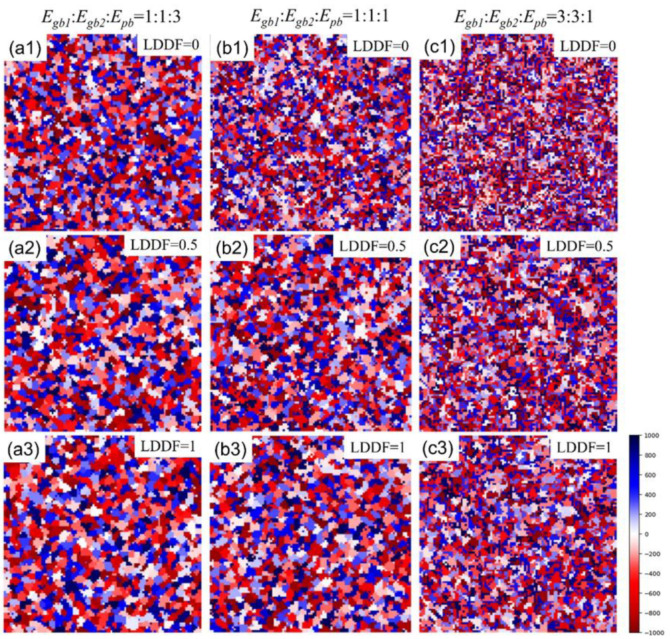
Two-dimensional simulation results after 10 MC steps of microstructure under the condition that the volume fraction of the second phase is 0.56. The ratios of *E_gb_*_1_, *E_gb_*_2_, and *E_pb_* are 1:1:3 in (**a1**–**a3**), 1:1:1 in (**b1**–**b3**), and 3:3:1 in (**c1**–**c3**). LDDF ranges from 0 to 1.

**Figure 3:**
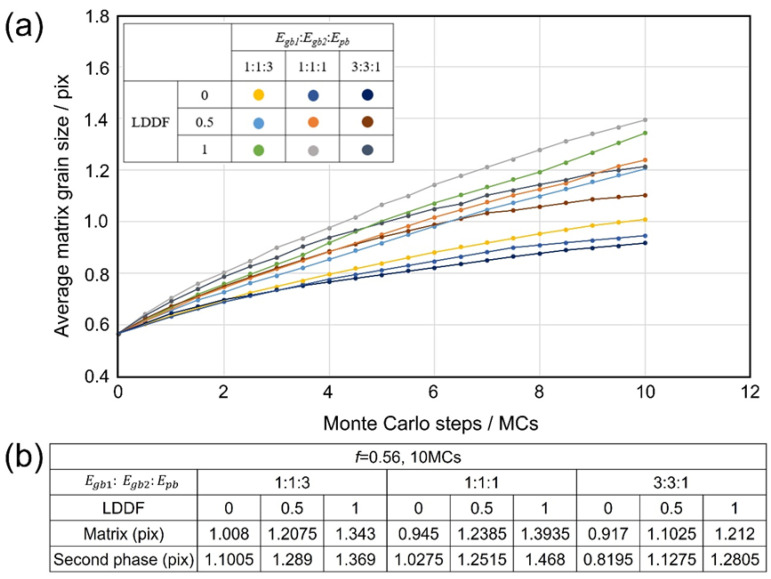
Two-dimensional simulation results of (**a**) the average matrix grain size as a function of MC steps and (**b**) the average grain size of the matrix and second phase after 10 MC steps on the condition that the volume fraction of the second phase is 0.56, with various ratios of *E_gb1_*, *E_gb2_*, *E_pb_*, and LDDFs.

Figure 2(a1–a3) shows the 2D simulation results of the microstructure after 10 MC steps under the condition that the volume fraction of the second phase *f* is 0.56, $E_{gb1}:\;E_{gb2}:E_{pb}=1:1:3$, and the LDDF is 0, 0.5, 1, respectively. Figure 2(b1–b3) shows the 2D simulation results of the microstructure after 10 steps under the condition that the volume fraction of the second phase *f* is 0.56, $E_{gb1}:\;E_{gb2}:E_{pb}=1:1:1$, and the LDDF is 0, 0.5, 1, respectively. Figure 2(c1–c3) shows the 2D simulation results of the microstructure after 10 steps under the condition that the volume fraction of the second phase *f* is 0.56, $E_{gb1}:\;E_{gb2}:E_{pb}=3:3:1$, and the LDDF is 0, 0.5, 1, respectively. It is obviously can be found that the two variations of $E_{gb1}:\;E_{gb2}:E_{pb}$ and LDDF significantly affect the microstructure evolution. At given ratios between grain boundary energy and phase boundary energy, the grain sizes of both the matrix and second phase also increase slightly with increasing LDDF, as shown in Figure 2(a1–a3,b1–b3,c1–c3). The microstructure, as shown in Figure 2(c1–c3), just displays the initial stage of grain growth to explore the affecting factors clearly and does not show the equilibrium state. Figure 3a shows the corresponding 2D simulation results of the average matrix grain size as a function of MC steps on the conditions of the volume fraction of the second phase 0.56, various ratios of grain boundary energy/phase boundary energy, and various LDDFs. With increasing MC steps, the average matrix grain size increases gradually and reaches an obvious state after 10 steps. The detailed simulated results after 10 MC steps are also summarized in Figure 3b. With increasing LDDF, the matrix and second-phase grain sizes increase under any proportion between grain boundary energy and phase boundary energy. Without consideration of the LDDF, the growth extent of the matrix and the second-phase grains decrease with the increasing ratio of grain boundary and phase boundary energy. Considering the LDDF and the value of 0.5, the growth extent of the second-phase grain decreases with the increasing ratio of grain boundary and phase boundary energy, while the growth extent of the matrix grain is maximum when the grain boundary energy equals the phase boundary energy. When the LDDF is 1, the growth extent of the matrix and the second-phase grain is maximum when the grain boundary energy equals the phase boundary energy. This is considered to be caused by grain growth decreasing the grain boundary and increasing the interface when the grain boundary energy is larger than the intergranular energy. When the grain boundary energy is greater than the phase boundary energy, the tendency of grain growth will be weakened. When the grain boundary energy is smaller than the phase boundary energy, the tendency of grain growth will be greater without consideration of the LDDF. Furthermore, the LDDF and the ratio between the grain boundary energy and the phase boundary energy may affect the growth behavior comprehensively.

**Figure 4:**
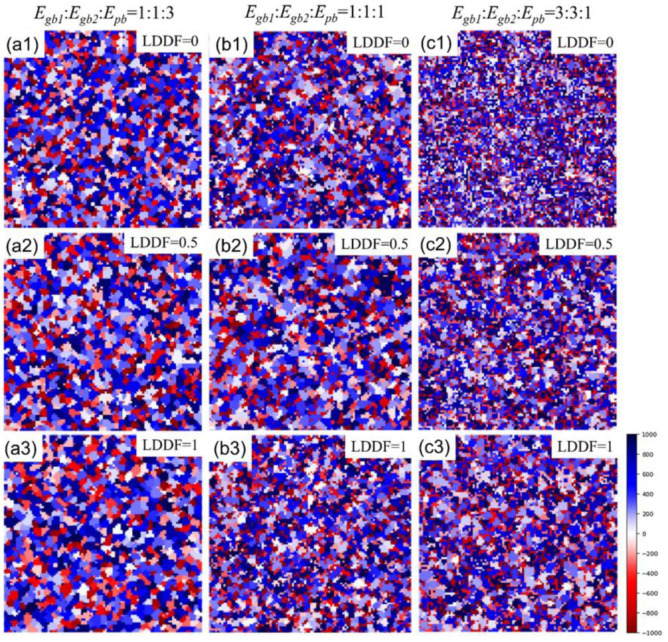
Two-dimensional simulation results after 10 MC steps of microstructure under the condition that the volume fraction of the second phase is 0.32. The ratios of *E_gb_*_1_, *E_gb_*_2,_ and *E_pb_* are 1:1:3 in (**a1**–**a3**), 1:1:1 in (**b1**–**b3**), and 3:3:1 in (**c1**–**c3**). LDDF ranges from 0 to 1.

**Figure 5:**
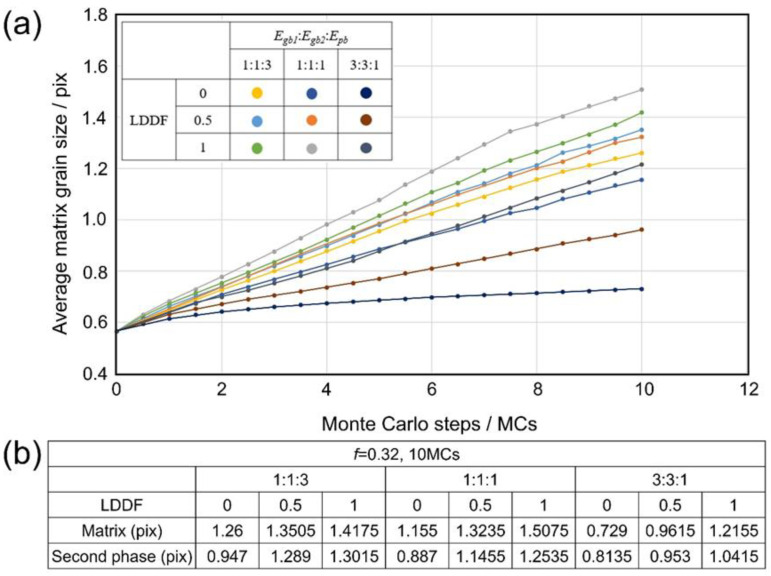
Two-dimensional simulation results of (**a**) the average matrix grain size as a function of MC steps and (**b**) the average grain size of the matrix and second phase after 10 MC steps on the condition that of the volume fraction of the second phase is 0.56, with various ratios of *E_gb_*_1_, *E_gb_*_2_, *E_pb_*, and LDDFs.

Figure 4(a1–a3) shows the 2D simulation results of the microstructure after 10 MC steps under the condition that the volume fraction of the second phase *f* is 0.32, $E_{gb1}:\;E_{gb2}:E_{pb}=1:1:3$, and the LDDF is 0, 0.5, 1, respectively. Figure 4(b1–b3) shows the 2D simulation results of the microstructure after 10 steps under the condition that the volume fraction of the second phase *f* is 0.32, $E_{gb1}:\;E_{gb2}:E_{pb}=1:1:1$, and the LDDF is 0, 0.5, 1, respectively. Figure 4(c1–c3) shows the 2D simulation results of the microstructure after 10 steps under the condition that the volume fraction of the second phase *f* is 0.32, $E_{gb1}:\;E_{gb2}:E_{pb}=3:3:1$, and the LDDF is 0, 0.5, 1, respectively. Similar to the previous case, the two variations of $E_{gb1}:\;E_{gb2}:E_{pb}$ and LDDF significantly affect grain growth behaviors. With increasing LDDF, the grain sizes of both the matrix and second phase increase slightly and correspondingly, as shown in Figure 4(a1), (a2), (a3), Figure 4(b1), (b2), (b3), Figure 4(c1), (c2), (c3), respectively. The microstructure, as shown in Figure 4(c1–c3), just displays the initial stage of grain growth to explore the affecting factors clearly and does not show the equilibrium state. Figure 5a shows the corresponding 2D simulation results of the average matrix grain size as a function of MC steps on the condition that the volume fraction of the second phase 0.32, with various ratios of grain boundary energy/phase boundary energy and LDDFs. The matrix grain grows with the increasing MC steps, and the average size after 10 MC steps varies with the conditions. The detailed simulated results after 10 MC steps are also summarized in Figure 5b. With increasing LDDF, the matrix and second-phase grain sizes increase under any proportion between grain boundary energy and phase boundary energy. When LDDF is 0 and 0.5, the growth extent of the matrix and the second-phase grains decrease with the increasing ratio of grain boundary and phase boundary energy. When LDDF is 1, the growth extent of the second-phase grains still decreases with the increasing ratio of grain boundary and phase boundary energy; however, the growth extent of the matrix grain is maximum when the grain boundary energy equals the phase boundary energy.

**Figure 6:**
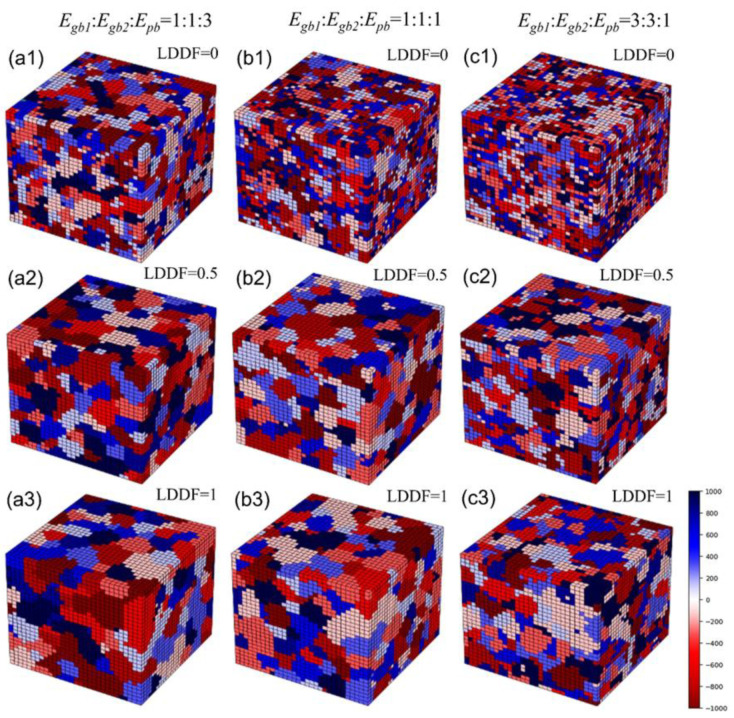
Three-dimensional simulation results after three MC steps of microstructure under the condition that the volume fraction of the second phase is 0.56. The ratios of *E_gb_*_1_, *E_gb_*_2,_ and *E_pb_* are 1:1:3 in (**a1**–**a3**), 1:1:1 in (**b1**–**b3**), and 3:3:1 in (**c1**–**c3**). LDDF ranges from 0 to 1.

**Figure 7:**
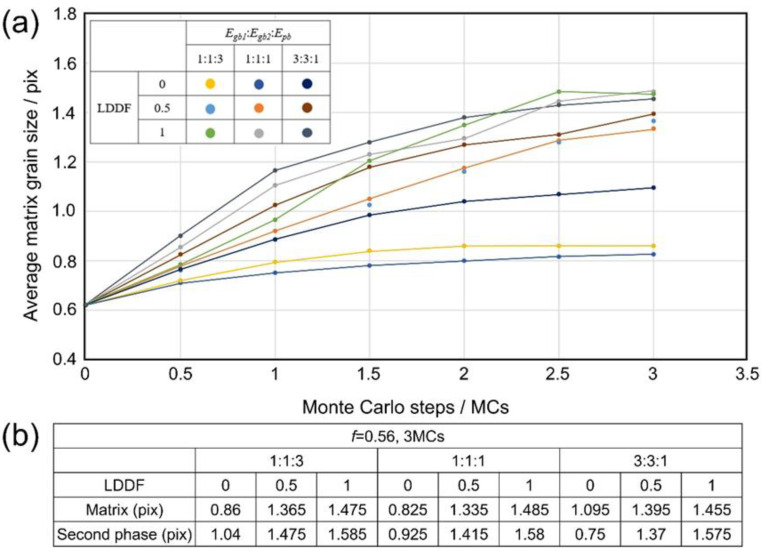
Three-dimensional simulation results of (**a**) the average matrix grain size as a function of MC steps and (**b**) the average grain size of the matrix and second phase after three MC steps on the condition that the volume fraction of the second phase 0.56, with various ratios of *E_gb_*_1_, *E_gb_*_2_, *E_pb_*, and LDDFs.

Figure 6(a1–a3) shows the 3D simulation results of the microstructure after three steps under the condition that the volume fraction of the second phase *f* is 0.56, $E_{gb1}:\;E_{gb2}:E_{pb}=1:1:3$, and the LDDF is 0, 0.5, 1, respectively. Figure 6(b1–b3) shows the 3D simulation results of the microstructure after 10 steps under the condition that the volume fraction of the second phase *f* is 0.56, $E_{gb1}:\;E_{gb2}:E_{pb}=1:1:1$, and the LDDF is 0, 0.5, 1, respectively. Figure 6(c1–c3) shows the 3D simulation results of the microstructure after 10 steps under the condition that the volume fraction of the second phase *f* is 0.56, $E_{gb1}:\;E_{gb2}:E_{pb}=3:3:1$, and the LDDF is 0, 0.5, 1, respectively. Similar to the previous case, the two variations of $E_{gb1}:\;E_{gb2}:E_{pb}$ and LDDF significantly affects grain growth behaviors. With increasing LDDF, the grain sizes of both the matrix and second phase increase slightly and correspondingly, as shown in Figure 6(a1), (a2), (a3), Figure 6(b1), (b2), (b3), Figure 6(c1), (c2), (c3), respectively. The microstructure, as shown in Figure 6(c1–c3), just displays the initial stage of grain growth to explore the affecting factors clearly and does not show the equilibrium state. Figure 7a shows the corresponding 3D simulation results of the average matrix grain size as a function of MC steps on the condition that the volume fraction of the second phase is 0.56, with various ratios of grain boundary energy/phase boundary energy and LDDFs. Regarding the LDDF effect on grain growth, the 3D simulation results are similar to those of 2D simulation. The detailed simulated results after three MC steps are also summarized in Figure 7b. With increasing LDDF, the matrix and second-phase grain sizes increase under any proportion between grain boundary energy and phase boundary energy. With increasing grain boundary energy, the average grain size of the second phase decreases gradually. When the grain boundary energy is greater than the phase boundary, the maximum average matrix grain size in the LDDF case is 0 and 0.5. When LDDF is 1, the average matrix grain size is maximum for higher phase boundary energy. The effect of boundary energy on the 3D simulation results of matrix grain growth behavior is more complicated than that of 2D simulation based on the different contributions of LDDF and boundary energy.

**Figure 8:**
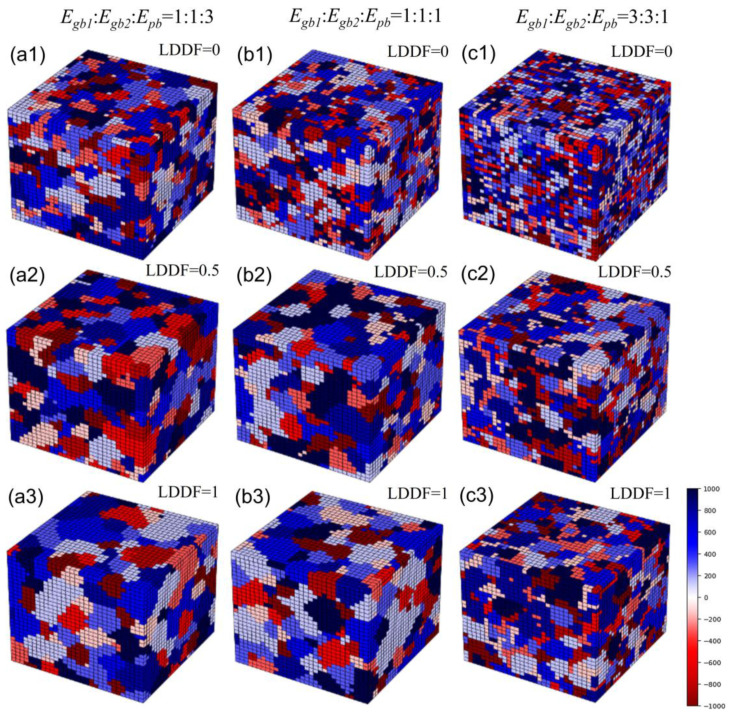
Three-dimensional simulation results after three steps of microstructure under the condition that the volume fraction of the second phase is 0.32. The ratios of *E_gb_*_1_, *E_gb_*_2,_ and *E_pb_* are 1:1:3 in (**a1**–**a3**), 1:1:1 in (**b1**–**b3**), and 3:3:1 in (**c1**–**c3**). LDDF ranges from 0 to 1.

**Figure 9:**
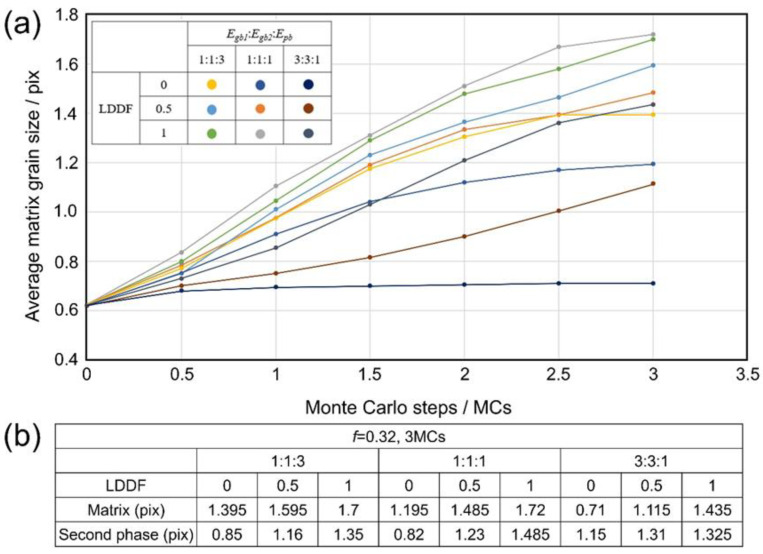
Three-dimensional simulation results of (**a**) the average matrix grain size as a function of MC steps and (**b**) the average grain size of the matrix and second phase after three MC steps on the condition that the volume fraction of the second phase is 0.32, with various ratios of *E_gb_*_1_, *E_gb_*_2_, *E_pb_*, and LDDFs.

Figure 8(a1–a3) shows the 3D simulation results of the microstructure after three MC steps under the condition that the volume fraction of the second phase *f* is 0.32, $E_{gb1}:\;E_{gb2}:E_{pb}=1:1:3$, and the LDDF is 0, 0.5, 1, respectively. Figure 8(b1–b3) shows the 3D simulation results of the microstructure after 10 steps under the condition that the volume fraction of the second phase *f* is 0.32, $E_{gb1}:\;E_{gb2}:E_{pb}=1:1:1$, and the LDDF is 0, 0.5, 1, respectively. Figure 8(c1–c3) shows the 3D simulation results of the microstructure after 10 steps under the condition that the volume fraction of the second phase *f* is 0.32, $E_{gb1}:\;E_{gb2}:E_{pb}=3:3:1$, and the LDDF is 0, 0.5, 1, respectively. Similarly, with increasing LDDF, the grain sizes of both the matrix and second phase increase slightly and correspondingly, as shown in Figure 8(a1), (a2), (a3), Figure 8(b1), (b2), (b3), Figure 8(c1), (c2), (c3), respectively. Figure 9a shows the corresponding 3D simulation results of the average matrix grain size as a function of MC steps on the condition that the volume fraction of the second phase 0.32, with various ratios of grain boundary energy/phase boundary energy and LDDFs. The detailed simulated results after three MC steps are also summarized in Figure 9b. With increasing LDDF, the matrix and second-phase grain sizes increase under any proportion between grain boundary energy and phase boundary energy. When the LDDF is 0 and 0.5, the average matrix grain size decreases when the grain boundary energy is greater. When the LDDF is 1, the average matrix and the second-phase grain sizes are maximum when the grain boundary energy equals the phase boundary energy. When the LDDF is 0 and 0.5 for the second-phase growth behavior, the greater grain boundary energy promotes second-phase growth.

Besides the above-mentioned affecting factors of boundary energy and LDDF, the volume fraction of the second phase still needs to be analyzed. With the increasing volume fraction of the second phase in 2D simulation, the average grain size of the second phase also increases, as shown in Figure 3b and Figure 5b. In addition, the average grain size of the matrix decreases when the grain boundary energy is equal to or is smaller than the phase boundary energy. When the grain boundary energy is greater than the phase boundary energy, with increases in the volume fraction of the second phase, the average grain size of the matrix increases in the case where LDDF is 0 or 0.5 and decreases in the case where LDDF is 1. In 3D simulation, according to Figure 7b and Figure 9b, with increases in the volume fraction of the second phase, the average size of the second phase increases while the average grain size of the matrix decreases when the grain boundary energy is equal to or smaller than the phase boundary energy. For greater grain boundary energy, the average grain size of the matrix increases with the increasing volume fraction of the second phase. Moreover, the average grain size of the second phase decreases when LDDF is 0 and increases when LDDF is 0.5 and 1.

**Figure 10:**
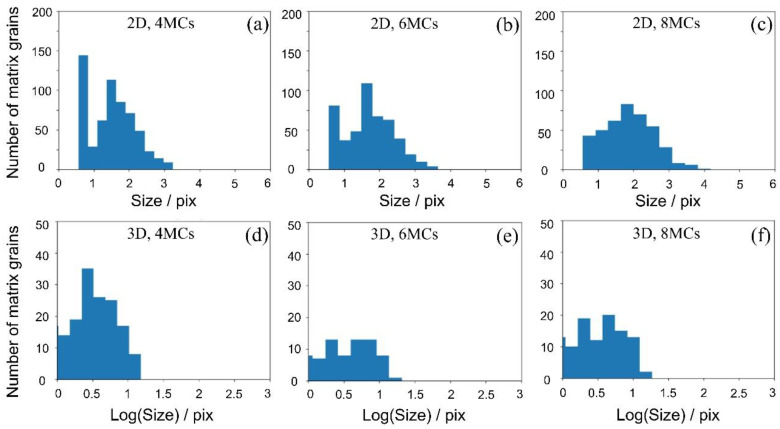
(**a**–**c**) Two-dimensional and (**d**–**f**) 3D simulation results of the distribution behavior of matrix grains under the condition that the volume fraction of the second phase *f* is 0.5, *E_gb_*_1_:*E_gb_*_2_:*E_pb_* = 1:1:1, LDDF is 1, and Monte Carlo steps are 4, 6, 8, respectively. The first left bar in each figure indicates the initial number of matrix grains at the beginning of simulation.

Figure 10a–f shows the 2D and 3D simulation results of the matrix grain distribution behaviors with time evolution on the condition that the volume fraction of the second phase is 0.5, $E_{gb1}:\;E_{gb2}:E_{pb}=1:1:1$, LDDF is 1, and MC steps are 4, 6, 8, respectively. The maximum size is 2.5~3 times larger than the average size for both 2D and 3D simulation results. The grain distribution behavior follows the Rayleigh distribution based on the 2D simulation results and the lognormal distribution based on the 3D simulation results. According to the 2D and 3D modeling and simulation, the grain distribution behaviors are consistent with previous studies [24].

The correlation of the grain size in a two-phase structure has been demonstrated [24,25]. In a two-phase microstructure, the matrix (*m*), the second phase (*s*), and their average grain sizes of the two phases ($\bar{R_{m}},\bar{R_{s}},\;\bar{R_{m}}+\bar{R_{s}}$) are the basic parameters. Here, the distribution of the matrix grain and the second phase are completely random. The matrix grain is adjacent to the second-phase grain randomly, forming a phase boundary. The volume fraction of the matrix and the second phase are $f_{m}$ and $f_{s}$. $S_{m/m},\;S_{s/s},\;S_{m/s}$ are the areas of the matrix/matrix grain boundary, the second phase/second phase grain boundary, and the matrix/second phase boundary. $S_{m}$ and $S_{s}$ are the total areas of the matrix and the second phase.

(5) $$S_{m/m}=S_{m}\cdot f_{m}=\frac{3V}{2}\cdot \frac{1}{\bar{R_{m}}}\cdot f_{m}^{2}$$

(6) $$S_{s/s}=S_{s}\cdot f_{s}=\frac{3V}{2}\cdot \frac{1}{\bar{R_{s}}}\cdot f_{m}^{2}$$

(7) $$S_{m/s}=S_{m}\cdot f_{s}+S_{s}\cdot f_{m}=\frac{3V}{2}\cdot \left(\frac{1}{\bar{R_{m}}}+\frac{1}{\bar{R_{s}}}\right)\cdot f_{m}f_{s}$$

The total interface energy

(8) $$E_{interface}=S_{m/m}\cdot E_{gb1}+S_{s/s}\cdot E_{gb2}+S_{m/s}\cdot E_{pb}$$

(9) $$\frac{\partial E_{interface}}{\partial \bar{R_{m}}}=\lambda \cdot \frac{\partial R}{\partial \bar{R_{m}}}$$

(10) $$\frac{\partial E_{interface}}{\partial \bar{R_{s}}}=\lambda \cdot \frac{\partial R}{\partial \bar{R_{s}}}$$

By substituting Equation (8) here, the correlation between particle sizes can be derived as follows.

(11) $$\bar{R_{m}}/{\left(f_{m}\cdot \left[\left(E_{gb1}/E_{pb}\right)\cdot f_{m}+f_{s}\right]\right)}^{1/2}=\bar{R_{s}}/{\left(f_{s}\cdot \left[\left(E_{gb2}/E_{pb}\right)\cdot f_{s}+f_{m}\right]\right)}^{1/2}$$

**Figure 11:**
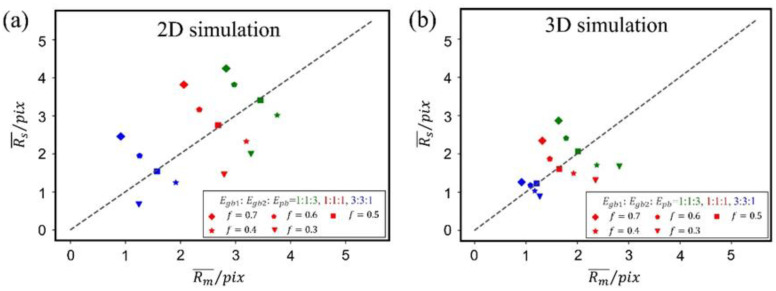
Relation between matrix grain size and the second-phase grain size based on (**a**) 2D and (**b**) 3D simulations under the condition of various volume fractions of the second phase *f*, ratios of *E_gb_*_1_, *E_gb_*_2_, *E_pb_*, LDDF is 0.5, respectively. The green, red, and blue colors indicate different grain boundary and phase boundary energy ratios.

According to the above correlations, Figure 11a,b shows the relation between the matrix grain size and the second-phase grain size based on 2D and 3D simulations under the condition of various volume fractions of the second phase, various ratios of *E_gb_*_1_, *E_gb_*_2_, *E_pb_*, and LDDF is 0.5, respectively. It can be found that the correlation is best fitted for both 2D and 3D simulations when the volume fraction of the second phase is 0.5. For other volume fractions of the second phase, the correlation goes away. A similar correlation can be obtained when the LDDF is 0 and 1. Here, the correlation of the grain size in a two-phase structure shows that the arrangement of grains in the matrix and the second phase is completely random, and the matrix grain is adjacent to the second-phase grain, forming a phase boundary. Considering that it is derived based on the assumption that the correlation is proportional to the volume fraction of each phase, the correlation was not satisfied in cases other than the volume fraction of the second phase being 0.5, which may result from the assumption of collapsed microstructure.

The length scale in the MC model can be converted to an absolute length scale in a simple linear manner by defining the grid point spacing λ as follows.

(12) $$\bar{R}=\lambda \bar{R_{MC}}$$

Here, $\bar{R}$ is the average size determined from the experimental results, and $\bar{R_{MC}}$ is the average size determined from the simulation results. There is a certain degree of freedom in setting the parameter λ, but it is the one that provides the spatial resolution of the simulation. The size is preferably smaller than the typical size of the particles being investigated so that the simulation can capture the shape and size of the particles at the required resolution.

Grain growth with time evolution in experiments and theory studies are expressed as follows.

(13) $${\bar{R}}^{n}-{\bar{R_{0}}}^{n}=k_{n}\cdot t$$

Here, $\bar{R}$ is the average size, $\bar{R_{0}}$ is the average when grain growth begins to progress steadily, *t* is the heating time.

In the MC algorithm, the time evolution of physical processes is modeled on the simulation time scale measured in the MC step. Therefore, the grain growth law in the MC simulation is expressed as the following equation.

(14) $${\bar{R_{MC}}}^{n}-{\bar{R_{0,MC}}}^{n}=k_{n,MC}\cdot t_{MC}^{\prime }$$

Here, $\bar{R_{MC}}$ is the calculated average grain size from the MC simulation results, $\bar{R_{0,MC}}$ is the initial size of the MC simulation, *n* is the power-law index, $k_{MC}$ is the grain growth rate, $t_{MC}$ is Monte Carlo step (MCs). $t_{MC}^{\prime }$ is the fitting result of $t_{MC}$ using the fitting parameter κ.

(15) $$t_{MC}^{\prime }=\kappa t_{MC}$$

From the theoretical equation of the grain growth law and Equation (13), the MC time $t_{MC}^{\prime }$ can be expressed as follows.

(16) $$t_{MC}^{\prime }=\frac{k}{\lambda^{n}k_{MC}}t$$

Here, λ is the value set earlier in the spatial scaling, and k and $k_{MC}$ are determined by regression analysis using grain growth experiment and simulation data, respectively.

**Figure 12:**
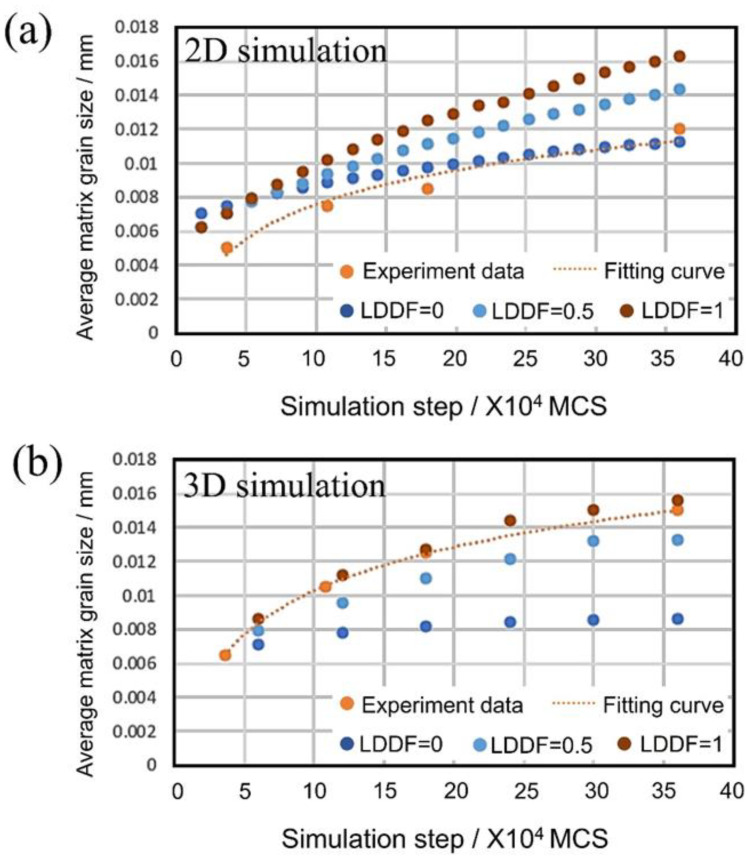
(**a**) Two-dimensional and (**b**) 3D simulation results of the average matrix grain size changes with MC steps under the condition that volume fractions of the second phase *f* = 0.56, and LDDF is 0, 0.5, and 1.

Figure 12a,b shows the 2D and 3D simulation results of the average matrix grain size changes with MC steps using the above value under the condition that volume fractions of the second phase *f* = 0.56, and LDDF is 0, 0.5, and 1, respectively. In the 2D simulation, when the LDDF is 0, the simulation result slightly deviates from the curve of the experimental results; considering the theory of grain growth in a two-phase structure, it is possible to obtain results that closely match the experimental results. This result is considered valid because the second phase cannot grow unless long-distance diffusion occurs. The 3D simulation result has become clear under these conditions where LDDF is 0.5 and 1, matching well with the experimental results.

### 3.2. Machine Learning

**Figure 13:**
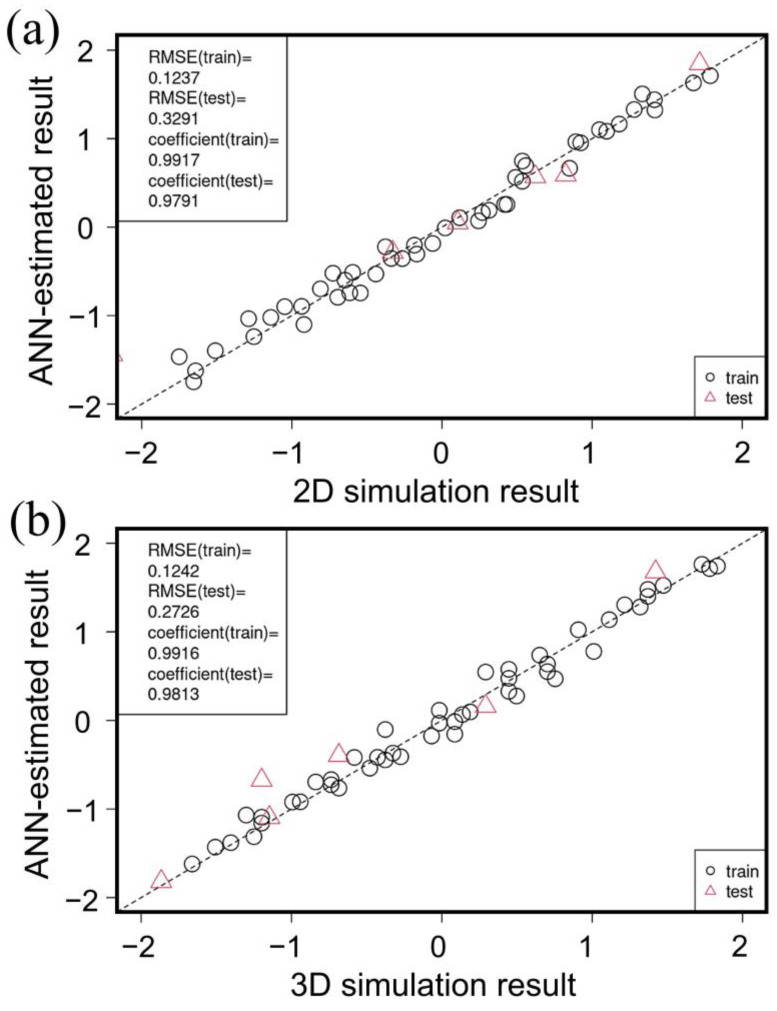
Correlations between (**a**) 2D (**b**) 3D simulation results and ANN estimated results.

In this study, we performed machine learning using simulation data to quantitatively evaluate the parameters that affect grain growth. The correlation between the 2D and 3D simulation results and the ANN forward estimated analysis results is shown in Figure 13a,b, respectively. The coefficient of determination is close to 1, and a relatively accurate model has been constructed. The results of the sensitivity analysis performed using the model constructed above are shown in Table 2. In the 2D simulation, the LDDF is the most important factor in grain growth, and in the 3D simulation, the second phase volume fraction is the most important factor in grain growth. From the above, it was possible to quantitatively analyze the influence of each parameter used in the experimental results, and it became possible to rank the influence of each parameter.

The temporal evolution and morphology of grain growth in 2D and 3D behaviors can be successfully simulated by the MC method to explore the affecting factors. Combining material informatics methods such as image engineering and simulation calculations to obtain microstructure descriptors based on the structural parameter–process–performance relationship obtained in the experiment and simulation. The affecting sensitivity of each descriptor can be obtained with high efficiency and precision through the analytic method of machine learning. Construct an optimized structural model through the inverse analytical method of machine learning, and determine the optimal parameters for the required structural performance. This research strategy has achieved practical application and effectively accelerates the design and development of advanced structural materials [26,27,28].

## 4. Conclusions

In this study, grain growth behavior in DP steel was studied by 2D and 3D MC modeling and simulation to estimate the effect of some key parameters involving matrix/matrix grain boundary energy, second phase/second phase boundary energy, matrix/second phase boundary energy, volume fraction of the second phase, long-distance diffuse frequency, and the Monte Carlo steps. The effects of each parameter on grain growth behavior were discussed, and it was found that both 2D and 3D simulations could reproduce the trends of the experimental results. When the grain boundary energy is greater than the phase boundary energy, grain growth can be suppressed. When the volume fractions of the matrix and the second phase were equal, the suppression of grain growth became obvious. The long-distance diffuse frequency can promote grain growth significantly. Machine learning was performed to conduct a sensitivity analysis of the affecting parameters and estimate the magnitude of each parameter’s effects on grain growth in the model. The simulation results obtain a better understanding of the affecting factors of grain growth behavior in DP steel. This method provides deeper insight into speeding up the material structure development of steel materials.

## Figures and Tables

**Table 1:** Some parameters used for 2D and 3D MC simulations.

Grain and phase boundary energy ratio (*E_gb_*_1_:*E_gb_*_2_:*E_pb_*)	1:1:3 1:1:1 3:3:1
Volume fraction of the second phase (*f*)	0.3~0.7
Long-distance diffusion frequency (LDDF)	0~1
Monte Carlo steps (MCs)	2D: 10 3D: 3

**Table 2:** Sensitivity analysis results of each parameter in 2D and 3D simulations.

	2D	3D
Volume fraction of the second phase (*f*)	0.90	2.94
Long-distance diffusion frequency (LDDF)	3.16	1.94
Matrix/matrix grain boundary energy (*E_gb_*_1_)	0.88	1.22
Second phase/second phase grain boundary energy (*E_gb_*_2_)	0.88	1.22
Matrix/second phase grain boundary energy (*E_pb_*)	0.16	1.05

## Data Availability

Data are contained within the article.
